# Observed Mealtime Interactions, Meal Healthfulness, and Childhood Obesity Among Low‐Income Families

**DOI:** 10.1111/ijpo.70125

**Published:** 2026-06-12

**Authors:** Hurley O. Riley, Karen E. Peterson, Katherine W. Bauer, Allison Choe, Julie Sturza, Katherine Rosenblum, Niko Kaciroti, Julie C. Lumeng, Alison L. Miller

**Affiliations:** ^1^ Department of Health Behavior & Health Equity School of Public Health, University of Michigan Ann Arbor Michigan USA; ^2^ Department of Nutritional Sciences School of Public Health, University of Michigan Ann Arbor Michigan USA; ^3^ Department of Environmental Health Sciences School of Public Health, University of Michigan Ann Arbor Michigan USA; ^4^ Department of Pediatrics, Michigan Medicine University of Michigan Ann Arbor Michigan USA; ^5^ Department of Psychiatry, Michigan Medicine University of Michigan Ann Arbor Michigan USA; ^6^ Department of Biostatistics School of Public Health, University of Michigan Ann Arbor Michigan USA

**Keywords:** childhood obesity, meal healthfulness, mealtime interactions

## Abstract

**Introduction:**

Family mealtimes are promoted as a strategy to address childhood obesity; however, the joint associations of different mealtime elements with childhood obesity are not often considered. The current study investigated how mealtime family functioning and the nutritional quality of meals separately and jointly associate with childhood obesity.

**Methods:**

Typical mealtimes for 273 children (*M* age = 5.9 years, SD = 0.7 years) were video‐recorded. Observed mealtime family functioning, specifically mealtime structure and behaviour management, was coded from the video‐recorded mealtimes. The nutritional quality of meals was assessed using the Healthy Meal Index (HMI).

**Results:**

Logistic regression models indicated no independent associations between mealtime family functioning or nutritional quality of meals and child obesity status. However, the nutritional quality of meals moderated the association between mealtime structure and child obesity status (Interaction beta −0.06, SE 0.03; *p* = 0.04). Stratified analyses indicated that greater mealtime structure was associated with a higher likelihood of obesity among children served low‐nutritional‐quality meals (OR = 4.17, 95% CI 1.13–15.50, *p* = 0.03).

**Conclusions:**

Greater mealtime structure may be associated with an increased risk of childhood obesity when meals have low nutritional quality. Thus, various mealtime elements may interact in association with childhood obesity.

**Abbreviations**
κ: kappa statistic
BMI: body mass index
MICS: mealtime interaction coding system

## 1 Introduction

Family mealtimes have been promoted as a potential strategy to address childhood obesity [1, 2, 3], but it remains unclear which aspects of family mealtimes protect against obesity [4]. Thus, a more in‐depth characterisation of family mealtimes in relation to childhood obesity may enhance our understanding of how mealtimes may, or may not, shape childhood obesity. For example, mealtime family functioning, or the way in which families manage and interact during a meal, has been studied in association with child weight outcomes, with the hypothesis that well‐functioning family mealtimes (e.g., clearly defined roles, adaptable, and positive communication) are associated with more optimal weight outcomes [5]. As others have highlighted [6], it may be particularly helpful to consider how elements of mealtime family functioning and the nutritional quality of family meals combine to associate with child weight outcomes. Using naturalistic observations to examine these factors can provide nuanced insights about a family's mealtime, capturing both mealtime dynamics and the nutritional quality of meals within the home [7]. Thus, the current study investigated whether observed mealtime family functioning and the nutritional quality of meals are separately and jointly associated with child obesity status.

Mealtime family functioning is hypothesised to be associated with child weight outcomes because social processes during meals (e.g., parents managing their child's eating) can directly affect child eating behaviours [8]. One dimension of mealtime family functioning, task accomplishment, is characterised by the family's capacity to maintain structure and continue the flow of the meal despite any disruptions [9]. Low task accomplishment during mealtimes may diminish the potential benefits of family meals, for instance, by leading families to feel more stressed as a result of the mealtime [10, 11]. Another dimension of mealtime family functioning, behaviour management, is characterised by how families express and maintain behavioural standards during meals [9]. Poor behaviour management may result in lower quality mealtimes, such as children being distracted or upset during meals, which can have negative implications for child eating [12]. Researchers have investigated mealtime family functioning in relation to child weight; however, studies have found inconsistent results, suggesting that task accomplishment and behaviour management may not directly associate with child weight outcomes [13, 14, 15]. This may indicate that other mealtime characteristics are also important to examine.

The nutritional quality of foods served during family meals is another mealtime element that may be associated with children's weight outcomes. A meta‐analysis found that higher food quality (e.g., more vegetables; less fast food) served during family meals was associated with lower child BMI [16]. The nutritional quality of family meals may influence how mealtime family functioning is associated with a child's risk of obesity [17]. For example, when less healthy foods are present during the mealtime, families who do not effectively manage child behaviour during mealtimes may yield to demands for palatable foods, allow children to reject healthy foods, and may not set limits on foods that should be consumed in moderation [18]. Thus, it seems likely that both mealtime family functioning and the nutritional quality of foods served during family meals are jointly associated with child weight outcomes. As few studies have looked at the combination of mealtime functioning and the nutritional quality of meals, more research is needed to parse out these mealtime characteristics in association with child weight outcomes.

The current study investigated whether observed mealtime family functioning—specifically mealtime task accomplishment and behaviour control—and the nutritional quality of meals are associated with child obesity status. We hypothesised that higher mealtime family functioning and serving meals with higher nutritional quality would be associated with a decreased likelihood of children having obesity. Further, the study investigated whether the nutritional quality of meals modified the association between mealtime family functioning and child obesity status. Investigating how these mealtime elements may interact in association with child obesity status will help provide a nuanced understanding of the association between family mealtimes and childhood obesity, which may help inform obesity interventions.

## 2 Methods

### 2.1 Participants

**FIGURE 1:**
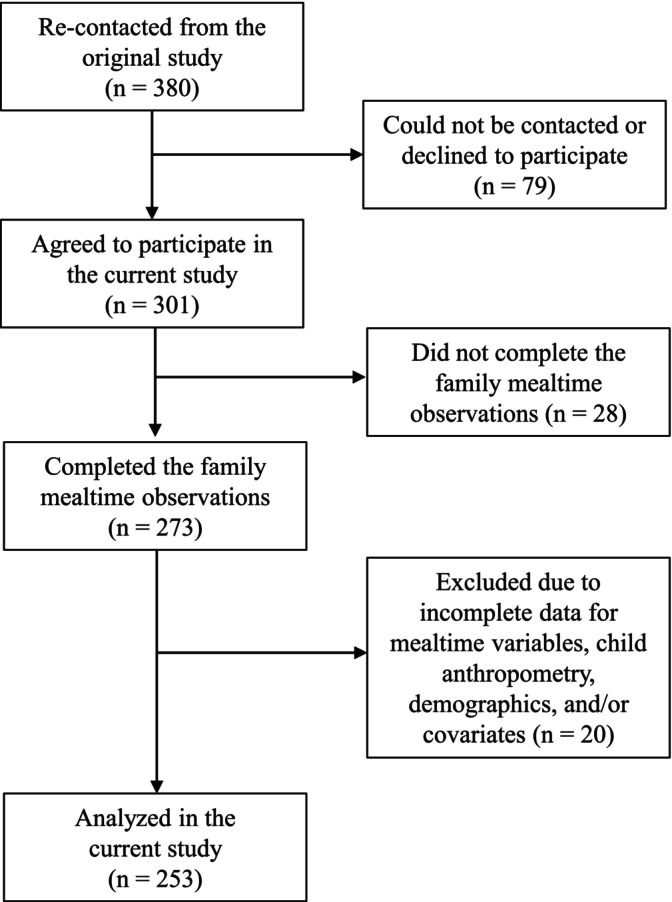
Flowchart of the analytic sample.

Study participants were primary caregivers residing in southcentral Michigan and their 4–8 year‐old children. Participants were originally recruited in 2009–2011 from Head Start, the federally‐funded preschool program for families living in poverty, to participate in a study of child eating behaviour. All families were living below the poverty line when their child was 4 years of age. Eligibility criteria included: caregiver is fluent in English and has less than a four‐year college degree; child was born at 35 weeks gestation or more without significant perinatal or neonatal complications; child does not have a history of food allergies or serious medical problems; and child is not in foster care. All 380 participants from the original study were re‐contacted by phone in 2011–2013 to recruit them for the current study focused on parental feeding practises (see Figure 1). Of the 301 (79.2%) caregiver/child dyads who agreed to participate in the current study, 273 dyads completed the family mealtime observations in the current study. The analytic sample (*n* = 253) was defined as those with complete data for mealtime variables, child anthropometry, demographic characteristics, and covariates. The analytic sample consisted of biological mothers (95%) and adoptive mothers, stepmothers, and grandmothers (5%); henceforth, we refer to these individuals collectively as ‘mothers’. All mothers provided written informed consent for themselves and their children, and were compensated $150 for participating in all study procedures. The University of Michigan Institutional Review Board approved this study (HUM00049008).

### 2.2 Study Procedure

Mothers completed two study visits conducted by trained research personnel. Research assistants administered questionnaires to mothers at the first visit to assess demographic characteristics and other parenting measures. Mothers then video‐recorded three naturalistic home mealtimes within 1 week of the first visit. Research assistants administered a post‐meal questionnaire to mothers after each meal in which they reported the foods and beverages available to the index child during the meal. The study concluded with a second study visit within a week at the child's Head Start center, where children's height and weight were measured according to standardised procedures.

### 2.3 Mealtime Observations

Mothers were loaned a video camera and asked to record the child's dinnertime on three weeknights (Monday–Thursday) over the next week, since weekends may be more variable and less typical. Mothers were instructed not to do anything special for these dinners because researchers wanted to understand what a typical dinner is like in different families. Mothers were asked to video‐record dinners that occurred when the mother was home and awake, the meal took place at home, and the meal was provided by the mother. If a language other than English was spoken at home, it was requested that the family speak only English during the video‐recorded meal. Mothers were instructed to set up the camera so the child's upper torso, plate, and drink were always in view, and to record the entirety of each meal. Mothers also completed a post‐meal questionnaire over the phone with a research assistant, where they reported the food and beverages available to the index child during the meal. Research assistants used this post‐meal questionnaire to code the healthfulness of meals across all three mealtimes after achieving reliability (*κ* > 0.70) with Registered Dietitians and nutrition doctoral students on a subset of 30 meals.

One mealtime observation video per index child, preferentially the second meal recorded, was selected for coding of mealtime family functioning. This approach resulted in 266 videos of the second mealtime observation being coded, and 6 videos of the first mealtime being coded (5 because the mothers recorded only one meal, and 1 because the child was not on camera during the second meal). Mealtime family functioning variables (i.e., task accomplishment and behaviour management) were coded by trained research assistants who achieved a reliability criterion of weighted *κ* > 0.69 on a subsample of 30 double‐coded videos.

### 2.4 Measures

#### 2.4.1 Mealtime Structure

The task accomplishment subscale of the Mealtime Interaction Coding Scale (MICS) was used to assess the quality of mealtime structure based on the mealtime recordings [19]. The task accomplishment subscale assesses the structural organisation and “flow” of mealtime, characterising the family's ability to achieve the goal of the index child having a dinner meal. Task accomplishment was rated on a 7‐point Likert scale ranging from 1 (complete disruption with lots of ‘missed opportunities’) to 7 (impressive effectiveness with active capitalization on opportunities). High task accomplishment scores reflect well‐managed and well‐planned meal routines that follow a clear structure (i.e., greater mealtime structure), whereas low scores reflect meals that are chaotic and do not show evidence of a regular routine (weighted *κ* = 0.73).

#### 2.4.2 Behaviour Management

The behaviour control subscale of the Mealtime Interaction Coding Scale (MICS) was used to assess how family members regulate their behaviour and the expectations for behaviour management during mealtimes. This may include disciplinary approaches to managing child behaviour (e.g., effectively addressing disruptions caused by children, such as disagreements about what or how much to eat). Behaviour control during the mealtime recordings was rated on a 7‐point Likert scale ranging from 1 (complete disruption with lots of ‘missed opportunities’) to 7 (impressive effectiveness with active capitalization on opportunities). High scores on behaviour control are characterised by capitalising on opportunities for positive interaction (e.g., teaching about manners) and demonstrations of respect for family members, whereas low scores are characterised by frequent and/or unresolved conflicts and disruptions during the meal, and either a chaotic/laissez‐faire or an overly rigid style (weighted *κ* = 0.69).

#### 2.4.3 Healthfulness of Meals

Healthfulness of meals (nutritional quality) was characterised from the post‐meal questionnaire using the Healthy Meal Index (HMI) [20], a validated tool for measuring the healthfulness of meals served to children. A meal's HMI Moderation score is based on the absence of four foods recommended to be consumed only in moderation: convenience foods, sugar‐sweetened beverages/diet drinks, added fats/saturated fats, and desserts. For each food item that is absent from the meal, 10 points are added to the Moderation score, resulting in a score range of 0–40. The meal's HMI Adequacy score is based on the presence of foods recommended for a healthy diet. Ten points are added to the Adequacy score for each type of food present: fruits, vegetables, dairy, and proteins. Five points are added to the Adequacy score for the presence of each component: quality vegetables, vegetable variety, grains, whole grains, and healthy fats, resulting in a total Adequacy score range of 0–65. The Moderation and Adequacy scores are summed to create a composite HMI Total Meal score. Higher HMI Total Meal scores indicate more healthful meals (i.e., lower presence of foods recommended to be consumed in moderation and/or higher presence of foods recommended for a healthy diet). The HMI Total Meal score for each of the three meals was averaged to determine the overall healthfulness of the meals.

#### 2.4.4 Weight Status

Research staff measured child height and weight without shoes or heavy clothing using a stadiometer and weight scale according to a standardised procedure [21]. Height and weight were collected twice, and the two measurements were averaged. If the two measurements differed by > 0.1 kg for weight or > 0.5 cm for height, two additional measurements were collected and averaged. Child BMI, adjusting for age and sex, was determined based on the US Centers for Disease Control and Prevention growth charts [22]. Children with a BMI ≥ 95th percentile for age and sex according to the US Centers for Disease Control and Prevention growth charts [22] were classified as having obesity. Each child's weight status was characterised as having obesity or not having obesity during analyses.

#### 2.4.5 Covariates

The following variables were measured and included as covariates in the current study: family income‐to‐needs ratio, whether a television was audible during the meal, and whether the mother ate the meal together with the child for any portion of the meal. Families' income‐to‐needs ratio was categorised using the midrange of the income range reported by mothers, adjusted for household size and rated relative to the federal poverty line. Using a coding scheme based on prior work [23], two research assistants coded whether the television was audible during the meal and whether the mother was eating with the child for any portion of the meal. The research assistants were trained to achieve reliability (*κ* > 0.7 for both codes), and 15 videos were coded by both raters to ensure reliability.

### 2.5 Statistical Analyses

Analyses were conducted using SAS 9.4 software [24]. Bivariate analyses were completed using *t*‐tests and ANOVA tests for categorical variables and Pearson correlations for pairs of continuous variables to determine which covariates would be included in the models. Logistic regression models examined whether mealtime family functioning variables and meal healthfulness are associated with child obesity status when controlling for covariates. To model joint effects, two separate logistic regression models were conducted to test whether each of the two mealtime family functioning variables interacted with meal healthfulness in association with child obesity status. Each model controlled for income‐to‐needs ratio, television audible during meal, and mother eating with child, as these variables were associated with key study variables. The mealtime family functioning variables and the HMI Total Meal scores were mean‐centered prior to modelling to aid the interpretation of the model results.

## 3 Results

### 3.1 Descriptives

Descriptive statistics of the sample and meal characteristics are presented in Table 1. On average, children were 5.9 years old (SD = 0.7) and 50.9% were male. Most mothers self‐identified as White, non‐Hispanic (55.3%), Black, non‐Hispanic (15.8%), Biracial, non‐Hispanic (16.8%), and Hispanic, any race (11.4%). The average income‐to‐needs ratio was 1.0 (SD = 0.6), indicating that families in the sample were living close to the poverty line, on average. No additional education beyond high school was reported by 47.2% of mothers. Mean maternal age was 31.2 (SD = 7.2). Video‐recorded meals lasted for 14.8 min on average (SD = 7.1).

**TABLE 1:** Characteristics of the sample (*n* = 273).

Variable	*N* (%) or mean (SD)
Child	
Age (years)	5.9 (0.7)
Sex	
Female	134 (49.1%)
Male	139 (50.9%)
Race/Ethnicity	
White, Non‐Hispanic	151 (55.3%)
Black, Non‐Hispanic	43 (15.8%)
Biracial, Non‐Hispanic	46 (16.8%)
Hispanic, any race	31 (11.4%)
Other, Non‐Hispanic	2 (0.7%)
Weight Status	
Underweight (BMI < 5th percentile for age and sex)	3 (1.1%)
Normal	154 (56.8%)
Overweight (BMI ≥ 85th percentile for age and sex)	54 (19.9%)
Obesity (BMI ≥ 95th percentile for age and sex)	60 (22.1%)
MICS Task Accomplishment	5.0 (0.7)
MICS Behaviour Control	4.9 (0.7)
HMI Total Meal Score	57.4 (10.3)
TV Audible During Meal	
Always Yes	77 (28.3%)
Always No	92 (33.8%)
Varied	103 (37.9%)
Father figure present during second mealtime observation	61 (22.3%)
At least one sibling present during second mealtime observation	181 (66.3%)
Mothers	
Age (years)	31.2 (7.2)
Education	
High school or less	129 (47.2%)
More than high school	144 (52.8%)
Income‐to‐Needs Ratio	1.0 (0.6)
Eating with Index Child During Meal	
Always Yes	161 (59.2%)
Always No	41 (15.1%)
Varied	70 (25.7%)

*Note:* We did not have all participants' data for child weight status (*n* = 271); TV audible (*n* = 272); income‐to‐needs ratio (*n* = 257); and eating with index child during meal (*n* = 272).

### 3.2 Logistic Regression Models

**FIGURE 2:**
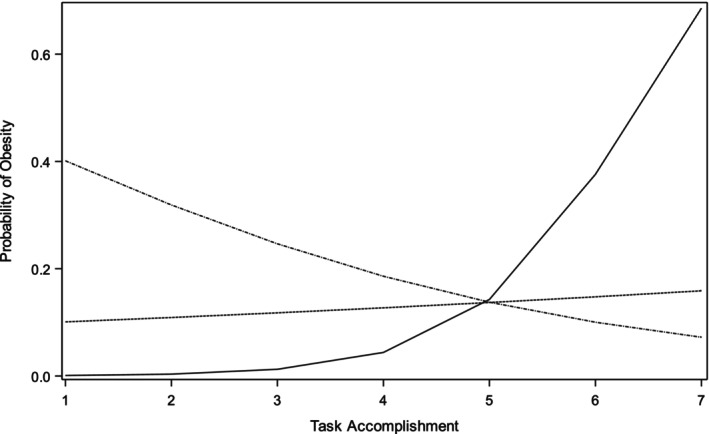
Predicted probability of child obesity varies by Task Accomplishment and HMI Total Meal Score. HMI Total Score Category (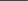: Low, < 25th percentile; 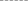: Average, 25th to 75th percentile; 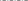: High, > 75th percentile).

Regression models investigating main effect associations indicated that task accomplishment (*β* = 0.25, SE 0.24; *p* = 0.30), behaviour control (*β* = −0.09, SE 0.22; *p* = 0.69), and HMI Total Meal Score (*β* = −0.01, SE 0.02; *p* = 0.38) were not associated with the likelihood of children having obesity. However, there was a significant interaction between task accomplishment and HMI Total Meal score in association with child obesity status after controlling for covariates (Interaction beta −0.06, SE 0.03; *p* = 0.04) (Table 2). As shown in Figure 2, after stratifying HMI Total Meal scores into low (≤ 25th percentile), average (> 25th percentile to < 75th percentile), and high (≥ 75th percentile) groups, greater task accomplishment was associated with an increased likelihood of children having obesity when families had a low HMI Total Meal score (OR = 4.17, 95% CI 1.13–15.50; *p* = 0.03), but not an average (OR = 1.03, 95% CI 0.53–2.00; *p* = 0.94) or high HMI Total Meal score (OR = 0.60, 95% CI 0.21–1.72; *p* = 0.34).

**TABLE 2:** Adjusted associations examining the interactions of mealtime family functioning (i.e., Task Accomplishment and Behaviour Control) with HMI Total Meal Score in association with child obesity status.

	*β* (SE)	*p*
Interaction model		
MICS Task Accomplishment	0.22 (0.25)	0.37
HMI Total Meal Score	−0.01 (0.02)	0.68
MICS Task Accomplishment X HMI Total Meal Score	−0.06 (0.03)	0.04
Income‐to‐Needs Ratio—low	−0.20 (0.23)	0.37
Income‐to‐Needs ratio—mid	−0.30 (0.27)	0.27
Income‐to‐Needs ratio—high (ref)	—	—
TV Audible During Meal—Always No	0.16 (0.23)	0.47
TV Audible During Meal—Varied	−0.12 (0.22)	0.58
TV Audible During Meal—Always Yes (ref)	—	—
Mother Eating with Child—Always No	0.36 (0.31)	0.26
Mother Eating with Child—Varied	−0.27 (0.27)	0.31
Mother Eating with Child—Always Yes (ref)	—	—
Interaction model		
MICS Behaviour Control	−0.14 (0.23)	0.55
HMI Total Meal Score	−0.01 (0.02)	0.60
MICS Behaviour Control X HMI Total Meal Score	−0.04 (0.02)	0.08
Income‐to‐Needs Ratio—low	−0.26 (0.23)	0.26
Income‐to‐Needs ratio—mid	−0.25 (0.27)	0.36
Income‐to‐Needs ratio—high (ref)	—	—
TV Audible During Meal—Always No	0.21 (0.23)	0.36
TV Audible During Meal—Varied	−0.17 (0.22)	0.44
TV Audible During Meal—Always Yes (ref)	—	—
Mother Eating with Child—Always No	0.26 (0.30)	0.39
Mother Eating with Child—Varied	−0.28 (0.27)	0.30
Mother Eating with Child—Always Yes (ref)	—	—

*Note:* MICS Task Accomplishment, MICS Behaviour Control, and HMI Total Meal Score were all mean centered prior to modelling.

Abbreviations: HMI = Healthy Meal Index; MICS = Mealtime Interaction Coding Scale.

There was no evidence of an interaction between behaviour control during mealtimes and HMI Total Meal score in association with child obesity status after controlling for covariates (Interaction beta −0.04, SE 0.02; *p* = 0.08).

## 4 Discussion

The current study examined whether mealtime family functioning, defined as mealtime structure (i.e., task accomplishment) and behaviour management, and nutritional quality, defined as meal healthfulness, were separately and jointly associated with child obesity status among a low‐income sample of families. Mealtime structure, behaviour management, and the healthfulness of meals alone did not associate with the likelihood of child obesity. This may reflect a lack of association; however, this may also reflect the importance of examining the joint associations of mealtime elements with child obesity status. Indeed, we found significant interaction effects. Specifically, greater mealtime structure was associated with a higher likelihood of children having obesity when families had a low HMI Total Meal score (i.e., served less healthy meals). These findings offer valuable insight into how mealtime characteristics may associate with childhood obesity.

There are several possible explanations for the observed findings. High mealtime structure may indicate that families place a high value on mealtimes. Yet, although valuing family mealtimes is generally encouraged, prioritising mealtimes that are characterised by low nutritional quality may undermine the potential benefits of family mealtimes [17]. A prior study, which assessed joint associations of family mealtime routines, meal planning, and meal healthfulness similarly found that 7–10 year‐old children in families with routine, planful, and healthful evening meals (i.e., serving less fast food and more fruits/vegetables) had a lower body fat percentage compared to children in families with unplanned, mixed healthfulness meals and families with planned meals that often served fast food [25]. Future work should consider joint aspects of the family mealtime—such as mealtime functioning and healthfulness—in association with child weight outcomes.

Behaviour management during mealtimes was not associated with child obesity status, as also observed in previous studies [14, 15]. It is possible that behaviour management during mealtimes is not associated with child obesity status. Of note, the code used in the current study and other studies [14, 15] captured overall behaviour management across the course of the meal, rather than specific food‐related incidents. In future research, it may be important to assess specific behaviour management strategies in relation to food and eating, such as responses to food tantrums. Furthermore, meal healthfulness did not modify the behaviour management‐obesity association. There may be additional variables that could act as modifiers that we did not examine. For example, a pilot study of low‐income families with preschool‐aged children observed racial/ethnic differences in behaviour management at mealtimes [26]. Another study found that mealtime conflicts may associate with weight for boys, but not girls [27]. Future research may benefit from examining such sociodemographic variables to understand more nuanced associations of behaviour management with child obesity status.

Establishing family mealtimes consistently inclusive of healthy nutritional options may be challenging for many low‐income families who have demanding jobs, conflicting obligations, and limited resources [28, 29]. Nevertheless, results from previous interventions have suggested that mealtime routines are modifiable [2, 30]. The HOME (healthy home offerings via the mealtime environment) Plus intervention, for example, targets family change in the planning, frequency, and healthfulness of family meals through group sessions and phone calls focused on nutrition education, meal planning, cooking skills, and reducing screen time. An evaluation of HOME Plus found that 8–12 year‐old children participating in the intervention improved their diet [31] and showed a modest decrease in excess weight gain [2]. Interestingly, HOME Plus was associated with lower BMI *z*‐scores among prepubescent [2], but not pubescent children, suggesting that early school‐age may be a critical period to intervene. Our study further suggests that it may be important not only to encourage well‐functioning family mealtimes, but also the serving of healthy foods during these mealtimes. Future research is needed, however, to understand which components of multi‐faceted interventions are effective and further, how to best support healthy family mealtimes among low‐income families, who may at times need to rely on ultra‐processed foods due to resource shortfalls [32].

A strength of the current study is that we observed family functioning during meals at home, which provided an objective measure of mealtime family functioning in a naturalistic setting. The MICS coding system is a well‐established measure, with demonstrated construct validity and interrater reliability [33]. Coding multiple mealtimes may have yielded a better sense of typical family functioning within families, but given time and resource constraints we were only able to code one mealtime recording. Some families may interact differently while a camera is present, so there could be an observer effect. Thus, we opted to code the second mealtime observation since families may be more acclimated to the camera by then. We were able to average the data on the healthfulness of meals across three typical dinner meals, which was an additional strength. As the Healthy Meal Index has only been validated with a low‐income sample [20], however, it is unknown how the healthfulness of meals in the current sample compares to meals by families with higher income. We did not have data regarding whether children with obesity were receiving any behavioural/lifestyle treatments during the study, which could have affected the mealtime. Other limitations include the cross‐sectional design, which limits the ability to infer causality, and that all participating families' children had attended Head Start preschools; thus, these results may not be generalizable beyond this population.

## 5 Conclusions

Mealtime family functioning was not associated with children's likelihood of having obesity among families with low income. Findings instead suggest that greater mealtime structure is associated with an increased likelihood of having obesity among children of families serving meals with lower nutritional quality. These results demonstrate that it is essential to consider the potential synergistic effects of multiple elements of family mealtimes, which can provide a more nuanced understanding of the association between family mealtimes and child weight outcomes.

## Author Contributions

Mr. Riley drafted and revised the initial manuscript, and approved the final manuscript as submitted. Dr. Peterson oversaw the development of the Healthy Meal Index by Kasper et al. [34], reviewed and revised the manuscript, and approved the final manuscript as submitted. Dr. Bauer reviewed and revised the manuscript and approved the final manuscript as submitted. Ms. Choe reviewed and revised the manuscript, and approved the final manuscript as submitted. Ms. Sturza conducted the statistical analyses and approved the final manuscript as submitted. Dr. Rosenblum conceptualised and designed the study, reviewed and revised the manuscript, and approved the final manuscript as submitted. Dr. Kaciroti reviewed the statistical analyses, reviewed and approved the final manuscript as submitted. Dr. Lumeng conceptualised and designed the study, reviewed and revised the manuscript, supervised the analyses, and approved the final manuscript as submitted. Dr. Miller conceptualised and designed the study, drafted and revised the initial manuscript, supervised the analyses, and approved the final manuscript as submitted.

## Funding

All phases of this study were supported by the National Institutes of Health (NIH), grant number R01HD061356.

## Conflicts of Interest

The authors declare no conflicts of interest.

## Data Availability

Research data are not shared.
